# Natural Gomesin-like Peptides with More Selective Antifungal Activities

**DOI:** 10.3390/pharmaceutics16121606

**Published:** 2024-12-17

**Authors:** Ilia A. Bolosov, Ekaterina I. Finkina, Ivan V. Bogdanov, Victoria N. Safronova, Pavel V. Panteleev, Tatiana V. Ovchinnikova

**Affiliations:** 1 M.M. Shemyakin & Yu.A. Ovchinnikov Institute of Bioorganic Chemistry, Russian Academy of Sciences, 117997 Moscow, Russia; bolosov@ibch.ru (I.A.B.); finkina@mail.ru (E.I.F.); contraton@mail.ru (I.V.B.); victoria.saf@ibch.ru (V.N.S.); p.v.panteleev@gmail.com (P.V.P.); 2 Moscow Center for Advanced Studies, 123592 Moscow, Russia

**Keywords:** antimicrobial peptide, gomesin, data mining, *Candida*

## Abstract

**Background**: Antimicrobial peptides are generally considered promising drug candidates for combating resistant bacterial infections. However, the selectivity of their action may vary significantly. Natural gomesin, isolated from haemocytes of the tarantula *Acanthoscurria gomesiana*, demonstrates a broad spectrum of antimicrobial activities, being the most effective against pathogenic fungi. **Methods**: Here, we searched for variants of natural gomesin-like peptides and produced their recombinant analogs in the bacterial expression system. The antimicrobial activities of the obtained peptides were tested against a panel of bacterial and yeast strains, and their toxicity towards human cells was examined. **Results**: Most of the new analogs of gomesin have primary structures homologous to that of the natural gomesin; however, they have fewer amino acid residues and post-translational modifications. One of the discovered analogs, the His-rich shorter peptide from the spider *Dysdera sylvatica*, designated as DsGom, displays antifungal activity comparable with that of natural gomesin. In the process of the structural–functional study of DsGom, it was shown that this analog retains a basic mechanism of action similar to that of natural gomesin. The DsGom analog has a significantly better toxicity profile as compared to gomesin. At the same time, the loss of the first Arg residue reduces, but does not annul, the antifungal activity of DsGom. Moreover, the acidification of the growth medium reduces the loss of the antifungal activity of this analog. **Conclusions**: The discovered natural gomesin-like peptides display more selective antifungal activities as compared to gomesin. The low cytotoxicity of DsGom, combined with its high antifungal activity and stability, allows us to consider it a promising drug candidate for the treatment of fungal infections, especially those caused by fungi of the Candida genus.

## 1. Introduction

*Candida* infections are a growing global public health problem, causing life-threatening diseases with high mortality. Patients hospitalized for COVID-19 are at risk for hospital-acquired infections (HAIs), including bloodstream infections and candidemia caused by *Candida* sp. [1]. Conventional antibiotics are gradually losing their effectiveness, thereby forcing the search for new sources of antimicrobial compounds. Among the compounds, which may become new prototypes of novel antibiotics, are antimicrobial peptides (AMPs), molecular factors of the innate immunity systems of vertebrates and invertebrates, as well as plants [2]. Of particular interest are β-hairpin AMPs, which have a rigid structure, often stabilized by one or more disulfide bridges. Their main advantage is structural resistance to protease degradation, which makes them promising as systemic drugs. As a rule, β-hairpin AMPs have a broad spectrum of activity; however, they also often exhibit cytotoxicity towards normal human cells [3].

Natural gomesin is a small, positively charged β-hairpin peptide consisting of 18 amino acid residues, including a pyroglutamic acid at the *N*-terminus, the *C*-terminal amide group, and four cysteine residues forming two disulfide bridges. Gomesin is produced in the hemocytes of the Brazilian spider *Acanthoscurria gomesiana* and exhibits a broad spectrum of activities against a panel of fungi and bacteria [4]. Besides antimicrobial activity, gomesin has been shown to exhibit cytotoxic activity against some types of cancer cells [5] and parasitic organisms, such as malarial plasmodium [6] and Leishmania [4]. It is generally accepted that the main mechanism of gomesin action is its ability to bind to negatively charged membranes, which is followed by their disruption [7]. However, upon the interaction of gomesin with a membrane, stable pore formation, characteristic of some other β-hairpin peptides, does not occur. It is believed that gomesin acts on membranes by a carpet mechanism [8]. Since the gomesin effect on a membrane is non-specific, gomesin exhibits a side cytotoxic activity against normal human cells and, in particular, has hemolytic activity. A number of studies have shown that gomesin exhibits a moderate hemolytic activity of about 40% at high concentrations of >100 µM [4,9,10]. Therefore, despite its broad spectrum of activities, the width of its therapeutic window significantly limits the potential of gomesin for therapeutic application.

Displaying multivarious biological activities, including antibacterial activity, anticancer activity, and antiparasitic activity, gomesin is of the greatest interest as an antifungal drug candidate. A number of studies have demonstrated the high effectiveness of gomesin against clinically significant yeasts, such as *Candida albicans* and *Cryptococcus neoformans* [4,9,11]. Notably, the activity of gomesin against fungi generally exceeds its activity against bacterial strains, which may indicate its key biological role. In addition, an in vivo study of gomesin on a murine model of candidiasis caused by a clinical isolate of *C. albicans* was carried out [12]. The study showed that gomesin effectively reduced fungal burden as compared to the control group. The antifungal effect of gomesin was observed at lower concentrations than that of fluconazole. Furthermore, a synergistic effect was shown when both compounds were used in combination. Since gomesin’s antifungal activity exceeds its activity against other targets, this allows us to consider this AMP a promising antifungal drug candidate.

The natural gomesin scaffold is of great interest, stimulating researchers to improve its properties. Several studies have focused on modifications of gomesin, changing specific amino acids or cyclizing the polypeptide chain [5,9,13,14]. However, despite the large number of studies, the molecular details of gomesin’s mechanism of action remain unclear. Furthermore, the diversity of natural gomesin analogs has been poorly studied so far. In this work, searching for new natural gomesin-like peptides and the study of their structure–functional features were carried out.

## 2. Materials and Methods

### 2.1. Bioinformatic Search and Biotechnological Production of Gomesin-like Peptides

TBLASTN software (https://blast.ncbi.nlm.nih.gov/Blast.cgi (last accessed on 12 September 2024)) was used to identify target genes for all available Araneae species in the whole-genome sequencing (WGS), the transcriptome shotgun assembly (TSA), the sequence read archive (SRA), and the expressed sequence tag (EST) GenBank databases, using the precursor protein of gomesin as well as a mature peptide sequence as a query with the values of the default parameters. Signal peptide sequences were identified with SignalP 5.0 (https://services.healthtech.dtu.dk/service.php?SignalP-5.0 (last accessed on 16 September 2024)). The obtained precursor proteins were also visually inspected to identify possible peptidylglycine α-amidating monooxygenase (PAM) processing sites. Finally, putative mature peptides were aligned with gomesin to classify them. The corresponding genes were optimized to be expressed in *E. coli* using the Codon Optimization Tool server (https://eu.idtdna.com/pages/tools/codon-optimization-tool (last accessed on 22 August 2024)). Recombinant AMPs were produced using an *E. coli* BL21 (DE3) heterologous expression system as C-terminal parts of fusion proteins containing an 8× His-tag, modified thioredoxin A (TrxA), and methionine residue for chemical cleavage [15]. Fragments encoding the studied peptides were assembled by overlap extension PCR and then ligated into expression plasmids and then used for cell transformation. The obtained transformed bacterial cells were grown in Miller LB broth (10 g/L of peptone, 5 g/L of yeast extract, 10 g/L of NaCl) containing 20 mM of glucose, 1 mM of MgSO_4_, 50 mM of Na_2_HPO_4_, 50 mM of KH_2_PO_4_, 25 mM of (NH_4_)_2_SO_4_, and 100 μg/mL of ampicillin. The cells were grown up to OD_600_ 1.0 and then were induced with 0.2 mM of isopropyl β-D-1-thiogalactopyranoside (IPTG). The culture was cultivated at 30 °C for 16 h under a rotation of 250 rpm. The primary purification of the peptides involved successive stages of ultrasonic cell lysis, immobilized-metal affinity chromatography (IMAC, Cytiva Life Sciences, Marlborough, MA, USA) of cell lysate, and CNBr cleavage of the fusion protein. Final purification was performed by high-performance liquid chromatography (HPLC) using a reverse-phase column, C18, of 5 µm, 120 Å, and 10 mm × 250 mm (GALAK Chromatography Technology, Wuxi, China). The purified peptides were analyzed using MALDI-TOF MS using a Reflex III mass-spectrometer and FlexAnalysis 3 software (Bruker Daltonics GmbH & Co. KG, Bremen, Germany).

### 2.2. Antimicrobial Assay

The antibacterial activity of the peptides was determined using two-fold serial dilutions in a sterile 96-well flat-bottom polystyrene microplate (Nest, Wuxi, Jiangsu, China) (Cat. No. 701011), as described previously [16]. In order to reduce the effects associated with the non-specific sorption of peptides by plastic, the experiments were carried out in the presence of 0.05% BSA solution. Activity against the studied bacterial strains (*Escherichia coli* DH10B (Invitrogen, Waltham, MA, USA), *E. coli* ML-35p, *E. coli* ATCC 25922, *Pseudomonas aeruginosa* ATCC 27853, *Staphylococcus aureus* ATCC 29213, and *Klebsiella pneumoniae* ATCC 700603) was measured in Mueller Hinton broth (MH) using a final cell concentration of 5 × 10^5^ CFU/mL. The antifungal activity of the peptides was assayed by the same method using final cell concentrations of 5 × 10^4^ or 2 × 10^4^ cells/mL in the case of *Saccharomyces cerevisiae* VKM Y1173 or fungi of the *Candida* genus (*C. albicans* ATCC 18804 and *C. albicans* ATCC 10231 and clinical isolates of *C. albicans* v47a3, *C. krusei* 225/2, *C. glabrata* 252/2, and *C. tropicalis* v13a4/2), respectively, as described previously [16]. Modified YPD (yeast extract: 5 g/L; peptone: 10 g/L; glucose: 10 g/L) liquid media or 0.5× Sabouraud broth were used for *S. cerevisiae* or fungi of the *Candida* genus, respectively. The assay results were analyzed after a 24 h incubation period. The viability of the microorganisms was assessed by adding of 0.1 mg/mL of resazurin (Sigma-Aldrich, St. Louis, MO, USA). After the addition of the redox indicator, the plate was incubated for an additional 2 h. The lowest concentration of the peptide at which no growth of the culture and no change in the color of the resazurin indicator was observed was used as the minimum inhibitory concentration (MIC). Fungal cell growth was also monitored using an inverted microscope and by measuring the optical density at 630 nm. Data are presented as median values from at least three independent experiments performed in triplicate.

### 2.3. Hemolysis and Cytotoxicity Assay

Hemolytic activity was determined using freshly isolated human erythrocytes (hRBCs) according to a previously published method [15]. Isotonic phosphate buffer (PBS) and 0.1% Triton X-100 were used as controls for the absence of lysis and complete disintegration of hRBCs, respectively. An α-helical membrane-active peptide, melittin, was used as a control drug, demonstrating a typical hemolysis curve.

The cytotoxic effect of the peptides was tested on human peripheral blood mononuclear cells (PBMC) using the resazurin assay. To perform the analysis, double serial dilutions of the studied peptides were performed in RPMI-1640 nutrient medium supplemented with 10% FBS in sterile 96-well flat-bottom polystyrene microplates (Nest, Wuxi, Jiangsu, China). Next, an equal volume of RPMI medium containing a suspension of freshly isolated PBMC was added to the peptide solution to a total volume of 100 μL and a cell concentration of 5 × 10^4^ per well. The plates were incubated for 20 h in a CO_2_ incubator (5% CO_2_, 37 °C) and then 5 µL of resazurin solution was added at a concentration of 0.1 mg/mL, after which the incubation was continued for 4 h. The fluorescence of the formed resorufin was measured using an AF2200 microplate reader (Eppendorf, Germany) using the following channel: λ_Exc_ = 535 nm; λ_em_ = 595 nm. The fluorescence signal in the wells containing cells cultured without peptides was taken as a 100% cell viability. Wells containing no cells were used as a negative control. Experimental data were obtained in two independent experiments performed in triplicate.

### 2.4. Bacterial Membranes Permeability Assay

The efficiency of the peptides in permeabilizing bacterial membranes was assessed using a classical approach using O-nitrophenyl-β-d-galactopyranoside (ONPG, AppliChem GmbH, Darmstadt, Germany) and the specialized *E. coli* strain ML35p, as described previously [16]. The signal level was measured at successive time intervals of 3 min from 0 to 57 min; then, the intervals were increased to 5 min up to 92 min and 30 min up to the final measurement at 272 min. The optical density (OD) of the solution rising due to the appearance of the hydrolyzed ONPG was measured at 405 nm using the AF2200 microplate reader (Eppendorf, Germany). The consistency of the observed curve pattern was confirmed across three independent experimental series; data from one of the representative series are presented.

### 2.5. Stability in Serum

The serum stability of DsGom was determined in 25% fresh human serum in PBS (pH 7.4), as described previously [17]. Briefly, 20 μL of aqueous peptide stock solution (5 mg/mL) was added to 480 μL of serum solution and incubated for 0, 2, 8, and 24 h at 37 °C. After incubation, serum proteins were selectively precipitated from the mixture by adding 10% TFA in the presence of 3.75 M of urea. Subsequently, the samples were stored at 0 °C for 30 min and centrifuged at 30,000× *g* for 10 min. Equal volumes of supernatants after serum protein precipitation were analyzed by analytical HPLC (Symmetry 300 C18 column, Waters, Milford, MA, USA) and MALDI-TOF MS. The amount of the intact peptide was estimated from the corresponding peak area on the HPLC chromatogram. For each peptide, two independent experiments were performed in triplicate. The comparison of the results and statistical processing were performed using GraphPad Prism 6.0 (GraphPad Software, Boston, MA, USA).

### 2.6. Assay by Flow Cytometry

Flow cytometry analysis was performed using a Novocyte 2060R flow cytometer (ACEA Biosciences Inc., San Diego, CA, USA), as described previously [18]. *C. albicans* ATCC 18804 at a final concentration of 2 × 10^4^ CFU/mL in 0.5× Sabouraud broth was used for analysis. Yeast-like cells were incubated in shaking conditions at 30 °C for 2 h in a volume of 4 mL in tubes pre-blocked with 0.1% BSA in the presence of gomesin or DsGom at concentrations of 0.25× MIC, MIC, and 4× MIC (0.097, 0.39, and 1.56 μM, respectively) or without the addition of the peptide. Treated cells and untreated controls were precipitated by centrifugation at 1600× *g* for 10 min, resuspended in 0.3 mL of PBS, and stained for 20 min at room temperature in the dark with propidium iodide (Biotium, Fremont, CA, USA) at a concentration of 4 μg/mL. The cells after thermal treatment at 99 °C for 10 min were used as a control of dead cells. The obtained data were processed using NovoExpress Software v. 1.2.4 (ACEA Biosciences Inc., San Diego, CA, USA).

## 3. Results and Discussions

### 3.1. Genome and Transcriptome Mining

**Figure 1:**
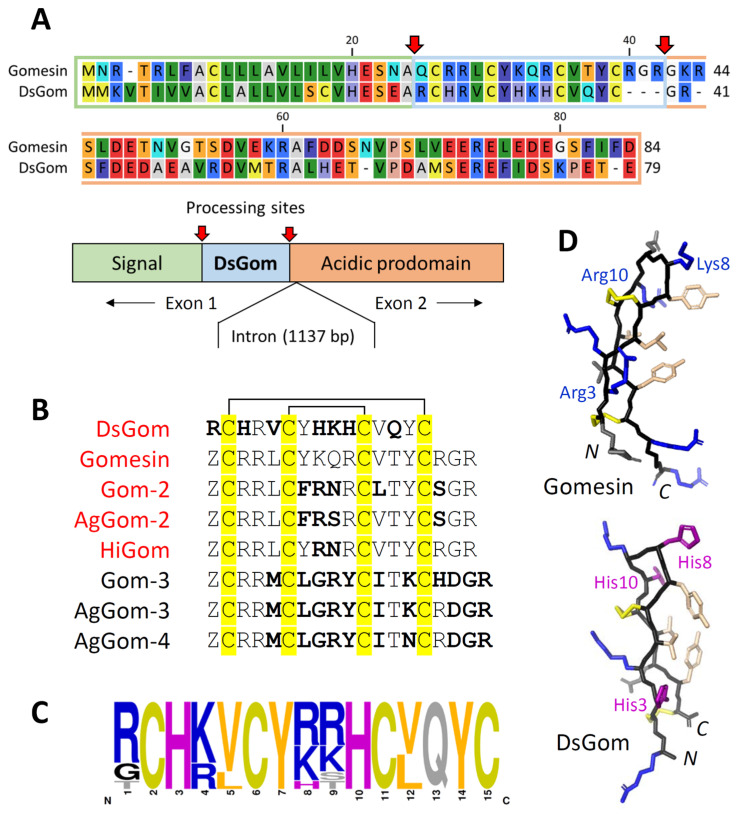
Structural analysis of the DsGom peptide and its precursor protein. (**A**) The amino acid sequence alignment of the precursor proteins of natural gomesin and DsGom. The alignment was made using the CLC Sequence Viewer software v8.0.0 (QIAGEN Aarhus Prismet, Aarhus, Denmark). The putative post-translational processing sites (signal peptidase and peptidylglycine α-amidating monooxygenase, respectively) are marked with red arrows. The presented data are based on the analysis of the WGS project of *Dysdera silvatica* (GenBank: QLNU02). (**B**) The amino acid sequence alignment of mature gomesin homologs from spiders (Gomesin, Gom 2 and 3 from *Acanthoscurria gomesiana,* AgGom 2, 3 and 4 from *Acanthoscurria geniculate*, and HiGom from *Hadronyche infensa*). Amino acid residues in the peptides that differ from those of natural gomesin are indicated in a bold font. The disulfide bonds are marked with square brackets and cysteine residues are marked in yellow. The peptides obtained in this study are colored in red. (**C**) Amino acid frequency in DsGom and its orthologs from spiders (*Dysdera* sp., *Heptathela* sp., *Ryuthela* sp., *Liphistius* sp., *Hypochilus* sp., and *Parachtes* sp.). The graph was plotted using the WebLogo server. (**D**) The spatial structure of gomesin in water (PDB 1KFP) and the top-rated modeled structure of DsGom predicted using the AlphaFold3 algorithm (https://alphafoldserver.com/ (last accessed on 23 August 2024)) with default parameters. Similarly to natural gomesin, disulfide bonds are formed according to the scheme C1–C4/C2–C3. The structures were visualized using the PyMOL v2.4.1 program (Schrödinger, Inc., New York, NY, USA).

The initial aim of this work was to identify natural variants of gomesin-like peptides in *Acanthoscurria* sp. As the result, five new isoforms were detected in the assembled transcriptomes and genomes of four species using the TBLASTN algorithm and by using the preprogomesin sequence as a query (Figure 1A, Supplementary Table S1). Interestingly, significant structural variability was observed in the β-turn region of the discovered peptides, but similarly to gomesin, the length of the peptides was 18–19 residues (Figure 1B). Our further efforts were aimed at finding shorter variants of the peptide. An extended search in all *Araneae* species led us to the discovery of a family of 15-residue gomesin-like peptides represented by infraorder Araneomorphae (*Dysdera* sp., *Parachtes* sp., *Hypochilus* sp.) and suborder Mesothelae (*Heptathela* sp., *Ryuthela* sp., *Liphistius* sp.). Analyses of all available databases (WGS, TSA, SRA) allowed us to identify 10 unique sequences in 25 different species (Supplementary Table S2). This family is also characterized by the presence of two conserved histidine residues at positions 3 and 10 of the polypeptide chain (Figure 1C). Moreover, one of the peptides, found both in the genome and transcriptome of *Dysdera sylvatica* and designated as DsGom, is characterized by the presence of three histidine residues in its structure (Figure 1B,D). The DsGom gene has two exons and precursor protein organization typical for gomesin-like AMPs, including the *N*-terminal signal sequence followed by the putative signal peptidase site, the mature peptide part followed by the putative peptidylglycine α-amidating monooxygenase (PAM) site, and the *C*-terminal 40-residue part enriched in negatively charged residues (Figure 1A).

### 3.2. Peptide Design, Expression, and Purification

In this work, we studied the discovered analogs of gomesin, most structurally similar to the natural AMP, including the previously described analog HiGom from *Hadronyche infensa* [19], as well as the revealed shortened peptide, designated as DsGom (Figure 1B). TrxA was used as a fusion partner to enhance the solubility of the resulting peptides and mitigate their toxic effects on bacterial cells [20]. The fusion protein was expressed in *E. coli* BL21 (DE3) cells, and the obtained total cell lysates were fractionated by affinity chromatography. After the purification and cleavage of the fusion protein, HPLC was used for the fine purification of the mature recombinant peptide. Natural analogs of gomesin contain the *N*-terminal glutamine residue. In natural peptides, modification of the *N*-terminal glutamine into pyroglutamic acid (5-oxoproline) occurs spontaneously or enzymatically by glutaminyl cyclase. To perform this modification in vitro, the purified gomesins containing the *N*-terminal glutamine was incubated overnight at 37 °C and a pH of 2.0, as proposed in [21]. The interconversion of the *N*-terminal glutamine into a cyclic form was confirmed by mass spectrometry.

### 3.3. DsGom Analog Has High Selectivity of Action Against Fungi

The antimicrobial activities of the obtained analogs of natural gomesin were tested against a panel of bacterial strains and yeast-like fungi. The results of the experiment are presented in Table 1.

The antibacterial activities of the discovered gomesin analogs were comparable to that of the natural gomesin. At the same time, all the studied analogs demonstrated a high level of activity against a panel of yeast-like fungi. Gomesin and its His-enriched analog DsGom demonstrated the most pronounced activity against the majority of fungal strains tested.

**Figure 2:**
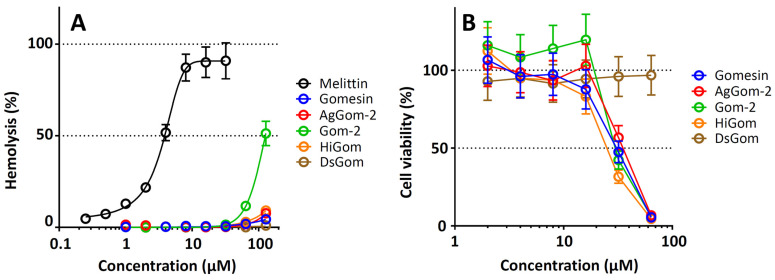
(**A**) The hemoglobin release assay of the gomesin analogs after 1.5 h of incubation and (**B**) cytotoxicity towards human PBMC after 24 h. Data are presented as the mean ± SD of two independent experiments.

The cytotoxic effects of the obtained analogs were studied using a hemolytic test against freshly isolated hRBCs, as well as against peripheral blood mononuclear cells (PBMC). The results are shown in Figure 2.

The hemolytic activity demonstrated by gomesin in the present work was lower than that described in previous works; however, this may have been due to the fact that a significantly higher concentration of hRBCs was used in the present experiment (4% vs. 0.4%) [4,9]. At the same time, the grade of the hemolytic activity of the full-length analogs of gomesin was comparable to that of gomesin, except that of the analog Gom2, which exhibited a significantly higher grade of hemolytic activity. Notably, the analog DsGom demonstrated an extremely low level of hemolysis. The grade of hemolysis at the concentration of 128 µM of this analog was ~1% (under the same conditions, the grade of hemolysis of gomesin was 5%). The increased hemolytic activity of the Gom-2 analog, however, did not lead to an increase in cytotoxic effects on PBMC (Figure 2B). All tested analogs, except for DsGom, demonstrated an average level of cytotoxic activities. The moderate toxicity of the natural gomesin analogs to normal cells is not surprising, since gomesin is often positioned as a peptide with anticancer activity [5,13,19]. It is interesting that the DsGom analog, as in the case of the hemolytic test, demonstrated an extremely low toxicity and ensured a survival of the PBMC of about 100% at the maximum peptide concentration. Thus, DsGom demonstrated an extremely high selectivity of the antifungal action.

### 3.4. The Mechanism of Action of the DsGom Analog Is Similar to That of Natural Gomesin

**Figure 3:**
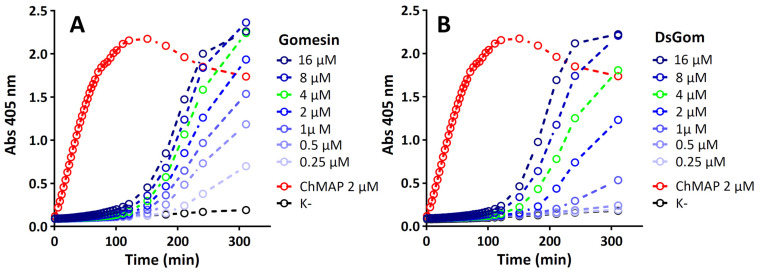
The effects of gomesin (**A**) and DsGom (**B**) at varying concentrations on the permeability of *E. coli* ML-35p membranes (ONPG assay). The concentration corresponding to the MIC value of each peptide against *E. coli* ML-35p is indicated in green. The α-helical membrane-active cathelicidin ChMAP was used as a positive control (marked in red).

In order to elucidate the mechanism of DsGom’s antibacterial action, we tested its ability to disrupt the integrity of *E. coli* ML35p membranes in comparison to natural gomesin using the chromogenic substrate ONPG for cytoplasmic β-galactosidase (Figure 3).

Both peptides showed similar kinetics of the permeability changes in the *E. coli* ML35p membranes. Unlike in the case of the control peptide ChMAP, demonstrating a complete membrane disruption at the concentration of 2 µM in less than 2 h, the effects of the gomesin analogs on the membranes became evident after more than 3 h. Thus, the gomesin analogs demonstrated slow kinetics of membrane permeability, which is similar to some other β-hairpin AMPs acting via the carpet mechanism [22].

**Figure 4:**
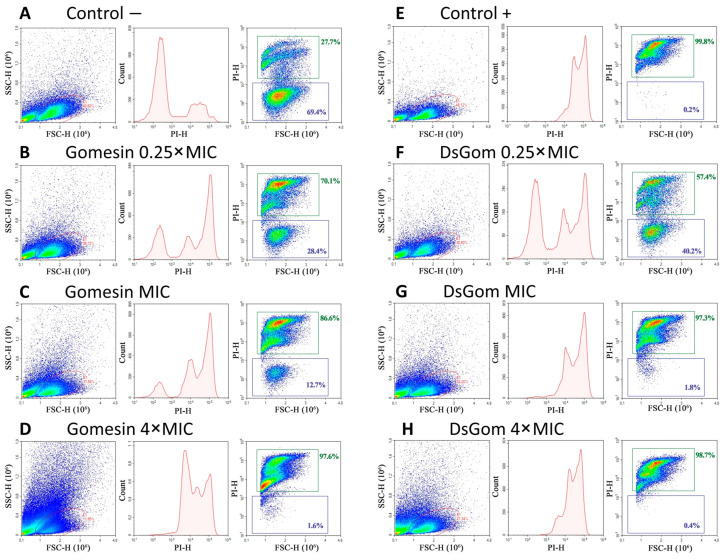
Flow cytometry analysis of cytotoxic effects exhibited by gomesin and DsGom on *C. albicans* ATCC 18804, measured by propidium iodide (PI) uptake. Live (**A**) and heat-killed (**E**) yeast-like cells were used as negative and positive controls, respectively. Gomesin (**B**–**D**) and DsGom (**F**–**H**) at concentrations of 0.25× MIC (**B**,**F**), 1× MIC (**C**,**G**), and 4× MIC (**D**,**H**) were used. Events on PI vs. count and FSC vs. PI plots are gated from FSC vs. SSC diagram.

Flow cytometry was used to compare the abilities of gomesin and its His-enriched analog DsGom to penetrate fungal cell membranes (Figure 4). For that purpose, *C. albicans* ATCC 18804 cells untreated and treated with peptides were stained with the red–fluorescent nucleic acid stain propidium iodide (PI), which can penetrate only dead cells with damaged membranes. Heat-killed yeast-like cells were also used as a positive control. In peptide-treated as well as heat-killed yeast-like cells, an increase in PI fluorescence was observed as a distinct shift in the peak along the *x*-axis (Figure 4B–H, PI vs. count diagrams). The percentage of PI-stained cells increased in the presence of both peptides at higher concentrations (Figure 4B–D,F–H, FSC vs. PI diagrams).

We revealed that both peptides effectively damaged fungal membranes, with more than 50% of cells being permeabilized even at the concentration of 0.25 × MIC (0.1 µM) after treatment for 2 h. Notably, 86.6% and 97.3% of PI-stained *C. albicans* cells were found at the MICs of gomesin and DsGom (0.4 µM in both cases), respectively, which indicated a rapid fungicidal effect of both peptides. A dramatic change in the morphology of part of the cell population was observed in the presence of gomesin at 4 × MIC (Figure 4D), possibly due to the full disruption of the fungal cells. At the same time, this effect was not observed in the case of heat-killed yeast-like cells and in the presence of DsGom at the concentration of 4 × MIC, which may indicate some difference in the mechanism of its action as compared to that of gomesin (Figure 4B–H, FSC vs. SSC diagrams). Thus, the mechanism of action of DsGom on yeast cells is maintained over a wide concentration range and is not associated with cell lysis, which, in general, correlates with its low cytotoxicity to mammalian cells and slow kinetics of bacterial membrane permeability.

### 3.5. DsGom Has a Very High Stability in the Presence of Serum

**Figure 5:**
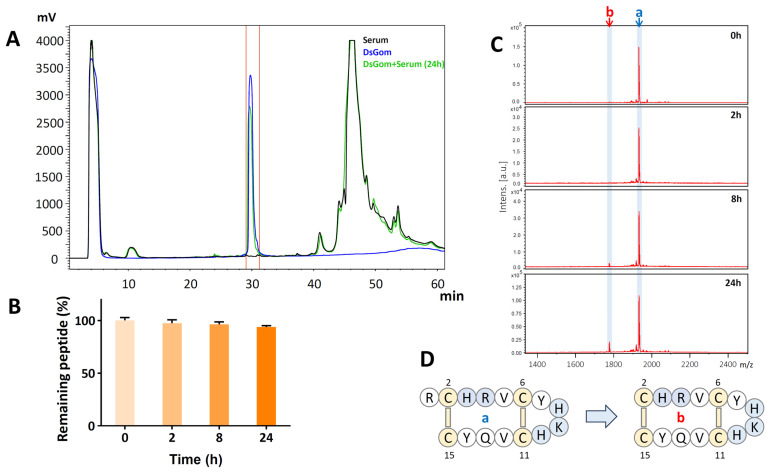
(**A**) HPLC analysis of the DsGom analog degradation by serum. The chromatograms of serum without the peptide (marked in black) and the peptide without serum (marked in blue) were used as controls. The figure shows a chromatogram after the incubation of DsGom with serum for 24 h (marked in green). The red lines indicate the peak area in the analysis. The area of the main peak was calculated and used to analyze the % of remaining peptide. (**B**) A diagram of the change in the remaining DsGom quantity depending on the incubation time with serum after 0, 2, 8, and 24 h. The HPLC peak area after 0 h of incubation is taken as a 100% remaining peptide. Analysis by the unpaired *t*-test method did not reveal a significant difference between the groups (estimated *p* value ≥ 0.32). (**C**) MALDI-TOF MS analysis of the target HPLC peaks containing DsGom and its shortened 14-residue analog after 0, 2, 8, and 24 h of incubation in 25% buffered human serum. (**D**) The cleavage of the *N*-terminal arginine residue by serum enzymes. The molecular masses of the DsGom natural peptide (a) and of the shortened 14-residue analog (b) are 1931 Da and 1774 Da, respectively, which matches well to the MALDI-TOF MS data.

A small size, a high antifungal activity, and a low toxicity make the DsGom analog promising as a drug candidate for the treatment of fungal infections. To examine the stability of the DsGom analog in serum, equal aliquots of the peptide were incubated for 0, 2, 8, or 24 h in PBS in the presence of 25% freshly isolated human serum obtained from a healthy donor. Then, the serum components were precipitated with TFA in the presence of urea, and the resulting supernatant containing the residual amount of the peptide was analyzed by the HPLC method. Previously, it has been shown that the gomesin scaffold is quite stable in the presence of serum enzymes, mainly due to two disulfide bonds [9,23]. Presumably, the DsGom analog has an even more rigid structure, being shorter and having fewer amino acid residues protruding beyond the cycle formed by the disulfide bonds. Thus, the DsGom analog might demonstrate a comparable or higher stability in the presence of serum. This thesis was confirmed by experimental results. Analysis of the chromatograms showed the absence of pronounced side peaks indicating the incomplete cleavage of the peptide (Figure 5A).

According to the results of the chromatographic analysis, after 24 h of incubation in the presence of 25% serum, at least 90% of the initial amount remained intact. The assessment of the residual peptide quantity by the unpaired t-test did not give a reliable difference between the groups without incubation and after 24 h of incubation. The analysis of the residual peptide quantity after incubation for 2 and 8 h showed significantly less difference compared to that of the control group (Figure 5B). The collected fractions corresponding to the DsGom peptide’s HPLC retention time were additionally analyzed using MALDI MS. Mass spectrometric analysis showed that after 8 h of incubation, the fraction corresponding to the DsGom peptide without the *N*-terminal arginine residue appeared in the main peak (Figure 5C,D). However, even after 24 h of incubation, the percentage of this form remained low (<10%). Thus, it can be concluded that DsGom, like natural gomesin, has outstanding stability in biological fluids, which makes it a promising candidate for use as an antifungal drug for topical or systemic application.

### 3.6. The Biological Activities of the DsGom Scaffold Has a High Tolerance to Amino Acid Replacements

We hypothesized that the His-enriched analog DsGom might change its spectrum of activities depending on pH of the medium, since this parameter significantly influences the peptide’s total charge. In order to confirm this hypothesis, as well as to conduct a study on structure–function relationships, we obtained a panel of mutant analogs of the DsGom peptide (Table 2). To test activities of the truncated peptide b (Figure 5D), resulting from the cleavage of the *N*-terminal arginine residue of DsGom in the presence of serum, we constructed the mutant analog DS1. In addition, we obtained a panel of mutant analogs with the sequential substitution of His residues with Arg or/and Lys (the analogs DS2 and DS5-7) in order to retain the total positive charge of the peptide but reduce potential pH sensitivity. Along with this, an inverse mutant analog was obtained with the Lys/His substitution in the middle of the polypeptide chain (the mutant analog DS4). In addition, a number of other mutant analogs were obtained. The analog DS3 (His8Gly) with a characteristic glycine residue in the β-turn region of known β-hairpin AMPs [24] was also obtained. Additionally, the mutant analog DS8, containing the triplet Lys-Gln-Arg, specific to natural gomesin, was obtained. The resulting panel of analogs was tested against the control strain *E. coli* DH10b, as well as against *S. cerevisiae* VKM Y1173 and *C. albicans* ATCC 18804 (Table 2).

As a result of testing, it was shown that the deletion of the *N*-terminal arginine led to a decrease in the antimicrobial activity of DsGom by two to four times as compared to the full-length peptide. Thus, partial degradation in serum does not lead to the complete inactivation of DsGom. Previously, the [2,3,4,5,6,7,8,9,10,11,12,13,14,15] gomesin fragment has also been shown to retain antimicrobial activity, with MIC values increasing two- to eight-fold as compared to the wild-type molecule [10]. Along with this, all other amino acid replacements did not have a significant effect on the antimicrobial activities of the mutant analogs of DsGom against the control strains.

**Figure 6:**
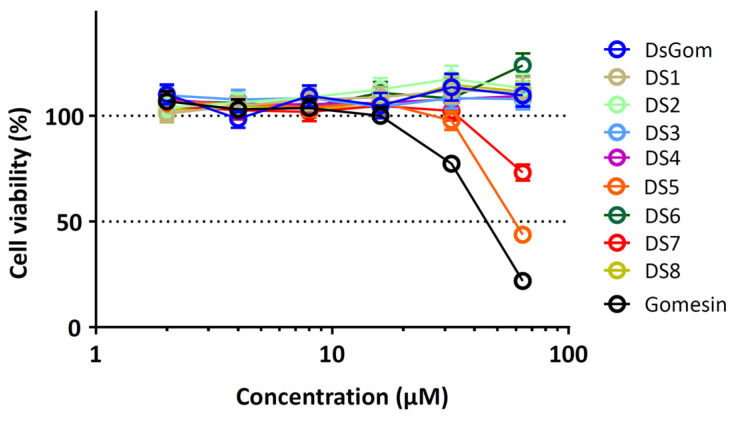
Cytotoxicity of DsGom mutant analogs towards human peripheral blood mononuclear cells (PBMC) after 24 h of incubation (resazurin assay). Data are presented as mean ± SD of three independent experiments.

The hemolytic activities of all the mutant analogs of DsGom did not have significant differences from that of the original peptide and were about 1% at concentrations up to 128 µM. However, the cytotoxic activities of the obtained panel of mutant analogs towards PBMC had differences (Figure 6). The replacement His3Arg by itself or in combination with the replacement His10Arg did not influence on the cytotoxic effects of the mutant analogs, while the His8Lys replacement significantly enhanced cytotoxic effects on PBMC (see graphs for the analogs DS5 and DS7). Interestingly, the introduction of the additional His9Gln replacement in order to reconstruct the Lys-Gln-Arg triad specific to gomesin masked the enhanced cytotoxic effect of the His8Lys replacement. We assume that the cytotoxicity-enhancing effects of all His8Lys-substituted analogs were due to the presence of two positively charged amino acid residues directly in the central β-turn region. However, it should be noted that all observed effects associated with cytotoxicity were evidenced exclusively at high concentrations of the peptide (>16 µM), which indicated that the non-specific cytotoxic effects of the mutants were amplified. At the same time, no significant effects of the introduced replacements on other biological activities of the mutant analogs were found. Our results from the ONPG assay and flow cytometry analysis suggest that the DsGom analog had a mechanism of action generally similar to that of natural gomesin. However, the short size, lower charge, and more favorable composition of amino acid residues in the β-turn region likely reduced non-specific interaction with membranes. Thus, there was a simultaneous reduction in antimicrobial activity and cytotoxicity against the hRBCs and PBMC.

### 3.7. Lowering the Medium’s pH Improves DsGom’s Selective Antifungal Activity

To check the hypothesis that the His-rich peptide DsGom is sensitive to the medium’s pH, we modified the experimental growth media by adding 0.005% TFA or 0.025% HCl. The acidification of the medium from the initial pH of 6–7 to a pH of 5.0 was reached. Since the pKa value for the imidazole ring of histidine is approximately 6, at a pH < 6, histidine is mainly positively charged, while at a pH > 6, it is mainly neutral [25]. We hypothesized that at a pH < 6, the His-rich mutant analogs of DsGom would increase their activity against the control strains, while the analogs with replacements of His for Lys or Arg would not be sensitive to the medium’s pH. In fact, the expected effect was not observed. When the test medium was acidified, an increase in activities was observed for all the studied analogs, which was on average two-fold, as compared to the non-acidified medium. Notably, the strongest effect of acidification of the medium was exerted with the DS1 analog, reducing its MIC values by at least four folds. Thus, acidic environmental conditions restored the antifungal activity of the DS1 analog, reducing its MIC to one comparable with the natural DsGom. As a result, we can conclude that a decrease in the medium’s pH generally increases the antifungal activity of DsGom and its analogs, which may be of interest when using the peptide in the complex treatment of vaginal candidiasis, especially together with suppositories reducing pH of the vaginal secretion [26].

## 4. Conclusions

In this work, we studied the natural diversity of gomesins and discovered a new family of gomesin-like AMPs consisting of 15 amino acid residues and enriched with His residues. The His-rich analog, designated as DsGom, exhibits moderate activity against bacteria and a high level of activity against fungi, similarly to natural gomesin. The main mechanism of action of the discovered analog is apparently also similar to natural gomesin. At the same time, DsGom has extremely low levels of cytotoxic and hemolytic activities, indicating its higher selectivity towards fungi. DsGom has a strong resistance to degradation in serum. Its fragment obtained by partial degradation retained the initial antifungal activity, especially at low pH values. Thus, the discovered DsGom peptide and its analogs are of great interest as a potential drug candidate for the treatment of fungal diseases, such as those caused by fungi of the *Candida* genus.

## Figures and Tables

**Table 1:** Minimum inhibitory concentrations (MICs) of gomesin analogs tested against bacteria and fungi.

Test Strain	Minimum Inhibitory Concentration (µM)				
	Gomesin	AgGom-2	Gom-2	HiGom	DsGom
Bacteria					
*S. aureus* ATCC 29213	16	>32	32	4	>32
*K. pneumonia* ATCC 700603	32	>32	32	32	32
*P. aeruginosa* ATCC 27853	4	>32	8	4	32
*E. coli* ATTC 25922	4	8	8	4	8
*E. coli* C600	4	4	4	2	2
*E. coli* ML-35p	4	4	4	2	4
*E. coli* DH10b	4	8	8	4	4
Fungi					
*S. cerevisiae* VKM Y1173	0.39	0.78	0.78	0.39	0.39
*C. albicans* ATCC 18804	0.39	0.78	1.56	0.39	0.39
*C. albicans* ATCC 10231	0.78	0.78	6.25	0.78	0.78
*C. albicans* v47a3	1.56	0.78	1.56	nd	nd
*C. krusei* 225/2	1.56	1.56	6.25	1.56	1.56
*C. glabrata* 252/2	0.78	1.56	1.56	1.56	0.78
*C. tropicalis* v13a4/2	0.78	0.78	1.56	nd	nd

nd—not determined.

**Table 2:** Minimum inhibitory concentrations (MICs) of DsGom mutants against bacteria and fungi.

Peptide		Minimum Inhibitory Concentration (µM)		
		*E. coli* DH10b	*S. cerevisiae* VKM Y1173	*C. albicans* ATCC 18804
DsGom	RCHRVCYHKHCVQYC	4	0.39	0.39
DS1	−CHRVCYHKHCVQYC	8	1.56	1.56
DS2	RC**R**RVCYHKHCVQYC	4	0.39	0.39
DS3	RCHRVCY**G**KHCVQYC	4	0.39	0.78
DS4	RCHRVCYH**H**HCVQYC	4	0.39	0.78
DS5	RC**R**RVCY**K**KHCVQYC	2	0.195	0.39
DS6	RC**R**RVCYHK**R**CVQYC	4	0.39	0.78
DS7	RC**R**RVCY**K**K**R**CVQYC	4	0.195	0.39
DS8	RC**R**RVCY**KQR**CVQYC	4	0.39	0.78

Amino acid residues in the peptides that differ from those of DsDom are indicated in a bold font, the deletion is marked with a minus sign.

## Data Availability

The original contributions presented in the study are included in the article/Supplementary Materials; further inquiries can be directed to the corresponding author.

## Supplementary Materials

The following supporting information can be downloaded at https://www.mdpi.com/article/10.3390/pharmaceutics16121606/s1, Table S1. Gomesin isoforms detected in the assembled transcriptomes and genomes using TBLASTN; Table S2. DsGom isoforms detected in the assembled transcriptomes and genomes using TBLASTN.
