# Supplementary material for: Natural Gomesin-like Peptides with More Selective Antifungal Activities
Source: Pharmaceutics. 2024 Dec 17;16(12):1606. doi: 10.3390/pharmaceutics16121606 (PMC11678162; doi:10.3390/pharmaceutics16121606)
Supplement: Supplementary file 1 [file pharmaceutics-16-01606-s001.zip › pharmaceutics-3355105-supplementary.pdf]

Supplementary Materials

Natural Gomesin-like Peptides with More Selective Antifungal Activities

Ilia A. Bolosov [1], Ekaterina I. Finkina [1,2], Ivan V. Bogdanov [1], Victoria N. Safronova [1], Pavel V. Panteleev [1,2] and Tatiana V. Ovchinnikova [1,2,*]

[1] M.M. Shemyakin & Yu.A. Ovchinnikov Institute of Bioorganic Chemistry, Russian Academy of Sciences, 117997 Moscow, Russia; bolosov@ibch.ru (I.A.B.); finkina@mail.ru (E.I.F.); contraton@mail.ru (I.V.B.); victoria.saf@ibch.ru (V.N.S.); p.v.panteleev@gmail.com (P.V.P.)

[2] Moscow Center for Advanced Studies, 123592 Moscow, Russia

[*] Correspondence: ovch@ibch.ru

Supplementary Table S1. Gomesin isoforms detected in the assembled transcriptomes and genomes using the TBLASTN.

Database	Animal	GenBank ID	Sequence	Cleavage Site
cDNA	*Acanthoscurria gomesiana*	DR443011.1	QCRRLCYKQRCVTYCRGR	GKR
cDNA	*Acanthoscurria gomesiana*	DR444611.1	QCRRLCFRNRCLTYCSGR	GKR
cDNA	*Acanthoscurria gomesiana*	DR444485.1	ECRRMCLGRYCITKCHDGR	GRK
TSA	*Acanthoscurria rondoniae*	GIOJ01043732.1	QCRRLCYKQRCVTYCRGR	GKR
TSA	*Acanthoscurria geniculata*	GAZS01063192.1	QCRRLCYKQRCVTYCRGR	GKR
TSA	*Acanthoscurria geniculata*	GAZS01063193.1	QCRRLCFRSRCVTYCSGR	GKR
TSA	*Acanthoscurria juruenicola*	GIIZ01049819.1	QCRRLCYKQRCVTYCRGR	GKR
TSA	*Acanthoscurria juruenicola*	GIIZ01049820.1	QCRRLCFRSRCVTYCSGR	GKR
TSA	*Acanthoscurria juruenicola*	GIIZ01055059.1	ECRRMCLGRYCITKCRDGR	GRK
WGS	*Acanthoscurria geniculata*	AZMS0104598094.1	ECRRMCLGRYCITNCRDGR	GRK
WGS	*Acanthoscurria geniculata*	AZMS0100164279.1	ECRRMCLGRYCITKCRDGR	GRK
WGS	*Acanthoscurria geniculata*	AZMS0100922710.1	QCRRLCYKQRCVTYCRGR	GKR
WGS	*Acanthoscurria geniculata*	AZMS0106884188.1	QCRRLCFRSRCVTYCSGR	GKR

Supplementary Table S2. DsGom isoforms detected in the assembled transcriptomes and genomes using the TBLASTN

Database	Animal	GenBank ID	Sequence	Cleavage site
WGS	*Dysdera silvatica*	QLNU02014176.1	RCHRVCYHKHCVQYC	GRS
SRA	*Dysdera silvatica*	SRX1612801-4	RCHRVCYHKHCVQYC	GRS
SRA	*Dysdera silvatica*	SRX1612801-4	RCHRVCYRKHCVQYC	GRS
SRA	*Dysdera longa*	SRX8629527	RCHRVCYRKHCVQYC	GRS
SRA	*Dysdera fustigans*	SRX8629533	GCHRVCYRKHCVQYC	GRS
SRA	*Dysdera crocata*	SRX566779	GCHRVCYRKHCVQYC	GRS
SRA	*Dysdera tilosensis*	SRX3777438	RCHRVCYRKHCVQYC	GRS
SRA	*Dysdera gomerensis*	SRX3777429	RCHRVCYRKHCVQYC	GRS
SRA	*Dysdera bandamae*	SRX3777421	RCHRVCYRKHCVQYC	GRS
SRA	*Dysdera verneaui*	SRX3777425	RCHRVCYHKHCVQYC	GRS
SRA	*Dysdera nesiotes*	SRX8629513	RCHRVCYRKHCVQYC	GRS
SRA	*Dysdera coiffaiti*	SRX8629488	RCHRVCYRKHCVQYC	GRS
TSA	*Dysdera* sp. IDV 7616	ICQP01018854.1	GCHKVCYRRHCVQYC	GRS
TSA	*Heptathela helios*	IAMB01034451.1	RCHKVCYKRHCVQYC	GRT
TSA	*Heptathela yanbaruensis*	IAWU01032890.1	RCHKVCYRRHCVQYC	GRT
TSA	*Heptathela yanbaruensis*	IBBQ01007806.1	RCHKLCYKTHCLQYC	GRT
TSA	*Heptathela higoensis*	IBNB01013561.1	RCHKVCYKRHCVQYC	GRT
TSA	*Heptathela kimurai*	ICFS01002070.1	RCHKVCYKRHCVQYC	GRT
TSA	*Heptathela amamiensis*	IBIH01022160.1	RCHKVCYKRHCVQYC	GRT
TSA	*Heptathela kikuyai*	IALQ01008812.1	RCHKVCYKRHCVQYC	GRT
TSA	*Heptathela yakushimaensis*	IALP01019820.1	RCHKVCYKRHCVQYC	GRT
WGS	*Ryuthela nishihirai*	JAWZYU010000007.1	RCHKVCYRRHCVQYC	GRT
TSA	*Ryuthela nishihirai*	IAIP01025855.1	RCHKVCYRRHCVQYC	GRT
TSA	*Ryuthela ishigakiensis*	IBLL01016278.1	RCHKVCYKRHCVQYC	GRT
TSA	*Ryuthela sasakii*	IAOR01024060.1	RCHKVCYKRHCVQYC	GRT
TSA	*Parachtes romandiolae*	ICQJ01009560.1	GCHRVCYRKHCVQYC	GRS
TSA	*Liphistius murphyorum*	ICAH01026570.1	RCHKLCYRSHCLQYC	GRG
RNAseq	*Liphistius malayanus*	ERX1795236	RCHRVCYRKHCVQYC	GRG
RNAseq	*Hypochilus* sp.	ERX1800235	RCHKVCYKRHCLQYC	GRS
RNAseq	*Hypochilus* sp.	ERX1800235	TCHKVCYKRHCLQYC	GRS
